# Digital justice and rights: bridging the fault lines in digital technology and SRHR

**DOI:** 10.1080/26410397.2025.2555645

**Published:** 2025-10-10

**Authors:** Priya Nanda, Nina Sun, Emma Pitchforth, Eszter Kismödi

**Affiliations:** a Independent Consultant, Gender and Global Health, New Delhi, India.; b Associate Editor, Sexual and Reproductive Health Matters, London, UK; c Executive Editor, Sexual and Reproductive Health Matters, London, UK; d Chief Executive, Sexual and Reproductive Health Matters, London, UK

**Keywords:** digital technology, sexual and reproductive health, human rights, digital justice

## Introduction

The unprecedented growth of digital innovations in the last decade has shaped recent discussions on health systems and health care delivery, including universal health care. Foreshadowing the immense possibilities of digital technology to enable the right to health is an apprehension that unregulated growth, without considerations of rights to equality, privacy, non-discrimination and accountability, can in fact weaken the impact on equality and universality of health care services, particularly for sexual and reproductive health and rights (SRHR). The UN Special Rapporteur on the Right to Health noted in 2023 that “digital innovation has strengthened the right to health for some, but warns it could enable violations and undermine this right”.^1^ The Special Rapporteur shared her concerns that digital technologies can perpetuate racism, sexism, ableism or discrimination based on sexual orientation or gender identity, among others.^1^ This concern would be amplified for SRHR that inherently face global and local, legal, normative, physical, financial and moral barriers to their actualisation, but evidence on this is lacking.

### The themed issue

This editorial reflects on key insights and evolving understanding from the themed issue on Digital Technology and Sexual and Reproductive Health and Rights. We examine how the evidence presented has shaped discourse around the complexities of digital health in the context of sexual and reproductive rights. The themed issue’s exploration of technology and healthcare intersections underscores both the opportunities and the persistent challenges in enabling equitable access to rights and services.

Previous *Sexual and Reproductive Health Matters* (*SRHM*) issues had captured both euphoric and cautionary perspectives on digital health for SRHR, particularly regarding self-care interventions. The present themed issue responded by presenting critical evidence on rights-based considerations related to the development, adoption and implementation of digital technologies for SRHR. The issue features research studies and commentaries examining how technology enables SRHR information to reach hard-to-access populations, links people to SRH services, provides access to pleasure-oriented sexual health information and facilitates care for communities that are otherwise stigmatised or hesitant to seek services in contexts of criminalisation.

The themed issue presents evidence-based analysis demonstrating how digital technologies have advanced the SRHR agenda, contributing to a discourse that extends beyond the widely acknowledged gender gap in access. The articles in this issue reveal a critical imbalance: while digital health has achieved remarkable success in enabling access to SRHR information and services, it simultaneously presents significant challenges to individual rights and social justice.

## Framing of rights and digital justice

Particularly over the past two years, a global discussion has emerged across various forums recognising that, regardless of how well-designed a digital system may be, it inevitably requires pathways to restorative and retributive justice to ensure unobstructed access to human rights.^2^ The concept of digital justice provides a crucial framework for situating discussions of digital harms and their effects on rights within the SRHR context.

Beyond protecting rights and preventing digital harms, digital justice emphasises the necessity of corrective actions to facilitate meaningful access to rights.^2^ Combined with a human rights framework, digital justice offers essential scaffolding for understanding digital health evidence. The themed issue demonstrates how inadequate protection of human rights and insufficient access to justice can readily overshadow digital health achievements in the SRHR domain. These concerns remain pressing and illuminate three critical areas that warrant careful examination: privacy and confidentiality; accountability for protecting data; and data justice and disclosure.

### Privacy and confidentiality

One of the most important SRHR use cases for digital health is the service provision for abortion, contraception and SRHR information and counselling in settings where these are limited, stigmatised or criminalised. Digitally provided information and services can be designed to be accessed with privacy, without stigma, conveniently and at low cost.^3,4^ Clients who use telemedicine for medical abortion, as evidenced from several of the articles in the themed issue, prefer seeking early abortion services in this manner, as it confers the most confidentiality and protects their privacy. Digitally provided information can also be designed to be pleasure-positive, especially for the population of younger users who otherwise have very censored or no access to SRHR information.^5^ Users of pleasure-positive content in settings where sex between minors is criminalised also need to be assured of privacy.

While the benefits are clear, the deficit of privacy considerations in the design and implementation of digital health technologies has been raised in various articles on this topic. The magnitude of personal health data on mobile platforms increases the risk of security breaches through hackers, as well as the risk that personal data could be sold to third parties for uses not originally consented to. Insufficient protection for digital health data can leave users vulnerable to criminalisation, stigma, discrimination and violence. In certain legal contexts, private companies and even public sector bodies can be compelled by law enforcement bodies to hand over personal data for criminal investigation purposes without sufficient safeguards, and can identify users of services that are restricted or even criminalised.^6^

For example, in their article on period-tracking apps, Kelly and Habib note that providers do not charge users money, *but* the apps often earn profits by selling users’ data to third parties, even if there is a promise of privacy advertised by the companies.^7^ While criticism over the monetisation of providing user data to third parties is not new, concerns regarding the disclosure of users’ reproductive health data in the USA have heightened since the overturning of *Roe v. Wade* in June 2022. The context of where and how the breach is happening can amplify vulnerability for the user multifold, and there is no redress to this concern. Some of the articles underscore that this is not only an issue of digital literacy for the user but also raises larger ethical concerns for technology firms and innovators.^7–9^

As part of a broader literature on digital privacy, there are special concerns regarding sexually marginalised groups like queer, youth and trans people, and sex workers in more criminalised settings. A study from Senegal on virtual sex work expands our understanding of digital security among sexually stigmatised populations.^10^ The advantages of virtual sex work, which include reduced exposure to sexually transmissible diseases and physical violence, online movement-building and new screening opportunities, need to be considered in the context of exposure to cyberbullying, censorship, harassment and image-based abuse. In Senegal, virtual sex work poses heightened risks due to cultural norms that place the burden of upholding family honour, or *sutura*, on women. For female sex workers, the potential erosion of this honour carries profound social consequences that are deeply internalised. Within this context, image-based abuse and cyberbullying compound their vulnerabilities, as such violations not only threaten personal safety but also risk public shame and familial disgrace. Building out from this article, and knowing that these scenarios are widespread, whether and how digital technologies can be used without backlash becomes an important parameter to understand their role in enabling sexual rights and agency.^10^ It is clear that technology has not been appropriately designed for all contexts and that it is necessary to distinguish between safe contexts and those that pose imminent threats from criminalisation or community stigmatisation.

### Accountability for protecting data

The lack of synchronisation between the growth of technology and privacy laws/regulations to protect health data is discussed in several articles. While digital surveillance is keeping pace with the widespread use of technology, the application of standards for regulation and protection is lagging behind. Although usually benign in terms of its intended use, technology that promotes expanded access to SRHR carries the highest risk of surveillance and misuse. The burden of protection seems to fall disproportionately on users to safeguard their digital health information. Often, data leak issues are beyond the control of the users and need to be guarded against through regulations. Authors of some of the articles that discuss data protection offer discrete suggestions at the level of the user, such as means to make it difficult to link personal user data with health data, or increased transparency around data-sharing options in the case of menstrual apps. But the realm of protection needs to go beyond the user. Lopes et al. advocate and provide a possible basis for a digital health governance agenda for SRHR.^11^ As Kelly and Habib note, FemTech mobile apps currently fall outside the scope of privacy protection laws.^7^ Thus there are provisions that can be made by firms to safeguard user data, underscoring the role of the industry in endorsing the privacy laws in this area.

Embedding data protection and clear consent into platform design emphasises a crucial paradigm shift.^7^ The General Data Protection Regulation (GDPR) – which applies to any organisation, regardless of location, that processes the personal data of European Union residents – mandates privacy rights pertinent to protecting users’ data, specifically the stipulation that the data controller, such as FemTech companies, present digital users with “easy-to-understand” language that includes a clear rationale for why that data is collected and how long it will be retained for, as well as instructions for users to request personal data erasure.

### Data justice and disclosure

Most articles in this themed issue underscore the critical importance of data privacy in protecting users’ personal information. Digital technologies for sexual and reproductive health and pleasure are increasingly interconnected across platforms, creating heightened vulnerability to data breaches. Information that appears secure on one platform may be readily exposed through vulnerabilities in another.

The degree to which users are aware of these data leaks – and how their information is subsequently utilised – transcends traditional concerns about data or digital literacy. This issue extends into complex domains of regulation and institutional responses to privacy violations, entering the emerging field of data justice. Within this framework, responsibility for corrective action shifts away from individual users and moves upward through the institutional chain, recognising that meaningful protection requires systemic, rather than individual, solutions.

As Albury et al. suggest, sextech can be extremely empowering to users, and its use should not be curtailed.^9^ Instead, efforts are needed to think through ethical frameworks within which sextech can and should be designed and developed such that consumers have full rights and disclosure on how their data is stored and used. Users need to be informed on how their data is collected, stored and used. There also needs to be a broader understanding that, while data is a public good to advance the applications of digital technology for sexual pleasure or for information and services, and can help to refine the user experience, companies and product developers must uphold standards of consent and privacy. Users should feel protected and know that there are governmental platforms for recourse and corrective action. Pillay et al. also highlight the care needed and the dangers of assumptions around understanding and meaning of consent for different parties and in different contexts.^12^

A comprehensive framework for digital justice in SRHR requires recognition of these three areas – privacy and confidentiality, accountability for protecting data, and data justice and disclosure – as interconnected and mutually reinforcing pillars. Each component strengthens the others: robust privacy protections enable meaningful accountability mechanisms, while clear disclosure practices enhance both user privacy and institutional responsibility. A truly effective digital justice framework must address all three dimensions simultaneously, as gaps in any one area can undermine the entire system of protection and empowerment for SRHR users.

## Evidence gaps and future research priorities

While the themed issue provides valuable insights into digital technologies for SRHR, several critical areas remain underexplored in the current evidence base, revealing significant gaps that warrant urgent research attention.

### Equity and inclusion for marginalised populations

There are notable gaps regarding the impact of digital technology on structurally excluded populations beyond sex workers. Research remains sparse on how digital SRHR technologies affect access to essential services among people who are HIV positive, people with disabilities, trans people and LGBQ+ communities. This absence highlights a fundamental concern: technology design often fails to centre the specific needs of marginalised groups, suggesting that the transformative potential of digital health may not reach those who need it most. This is particularly concerning for populations who face extreme stigma or are traditionally hard to reach, as existing systemic inequalities may be amplified rather than addressed through digital interventions. The dual nature of technology – as both supportive and restrictive – creates new paradigms for enabling equity and non-discrimination.

### Governance and regulatory frameworks

The evidence base lacks comprehensive analysis of good practices for developing rights-based laws, regulations and policies governing digital technologies for SRHR. Similarly, research examining the role of digital technology in organising and advocacy for SRHR movements remains limited, despite the growing importance of digital platforms in rights-based activism.

### Participatory design and meaningful engagement

A significant gap exists in evidence around meaningful engagement of key populations in the design and implementation of digital interventions. While “users” are often engaged through user experience (UX) design processes, it is done in a cursory manner. This represents a critical oversight, as participatory approaches are essential for ensuring digital technologies truly serve the communities they aim to support.

These evidence gaps represent important frontiers for advancing research that can inform more inclusive, rights-based approaches to digital health in SRHR.

## Looking to the future

Contradictions are inherent in the way technology has been rapidly developed for scale. On the one hand, there is reported misuse of data and violation of privacy, while on the other hand anonymised use of data from platforms is critical to innovation. An innovator needs data to advance the prowess of technology and make it work for diverse use cases. How to strike a balance between optimising data use while protecting the privacy of the user is a key area for regulation. The onus currently lies with the user, whose access is mitigated and restricted and who has to carefully navigate the digital space to leave few trails behind in contexts where services they seek are stigmatised or even criminalised. Yet it is clear from both the current global discourse and the articles in this themed issue that the discussion needs to shift to a demand for higher accountability and safeguards, as well as continuing to advocate for decriminalisation and non-discrimination. The evidence presented in this themed issue underscores the need to shift responsibility for digital privacy protection from individuals to companies and governments.

Digital technology can reproduce and amplify the existing fault lines within SRHR unless it is deliberately designed with distributed accountability for safeguarding users. This requires collaboration between regulators, developers and users, grounded in a shared understanding of and commitment to protecting individual digital rights. The insights in this themed issue provide a nuanced understanding of the problems and help to conceptualise rights and digital justice as an interconnected construct for understanding digital health interventions. Future scholarship and evidence should present a more thoughtful interrogation and insights on mitigation strategies and the role of the private and public sectors in protecting data misuse/leaks. There is a need for more research-driven insights on how to reduce risks through formal regulations and an emergence of promising practices in the SRHR use case, including perspectives on how user participation can help shape a more effective regulatory space. Since the launch of this themed issue, the pervasive spread of misinformation and disinformation has emerged as a significant concern and should be addressed in future *SRHM* calls and publications.

## Provenance statement

This article was commissioned and has been internally reviewed by editorial team members who are not coauthors.
